# Integrating climate scenarios and thermal welfare thresholds to project future heat stress risk in beef cattle

**DOI:** 10.1007/s00484-026-03217-1

**Published:** 2026-05-07

**Authors:** Giampiero Grossi, Andrea Vitali, Nicola Lacetera, Chiara Rossi, Nicola Lacetera

**Affiliations:** 1 Department of Agriculture and Forest Sciences, University of Tuscia, Via San Camillo De Lellis, Viterbo, 01100 Italy; 2 Department of Economic Sciences, University of Bologna, Piazza Scaravilli 2, Bologna, Italy

**Keywords:** Climate change, Shared socioeconomic pathways, Heat-Load Index, Heat stress categories, Meat production

## Abstract

Global demand for beef is projected to rise, coinciding with increasing climate change-related threats to animal welfare and productivity. Heat stress represents a major risk, impairing cattle health, growth, and reproductive efficiency. This study employed the Heat-Load Index, a composite measure of temperature, humidity, solar radiation, and wind speed, to classify heat stress into five categories. Using high-resolution climate projections from the NEX-GDDP-CMIP6 archive and a Multi-Model Ensemble of 27 global circulation models, we assessed heat stress risk for beef cattle worldwide under four Shared Socioeconomic Pathways (SSP1-2.6, SSP2-4.5, SSP3-7.0, SSP5-8.5) through 2100. Results indicated a progressive expansion of severe and extreme heat stress categories, particularly in tropical and subtropical regions, with increasing encroachment into temperate zones by the late century. These findings highlight the urgent need for climate-smart livestock strategies, including genetic selection, improved housing, and adaptive management, to safeguard animal welfare and sustain global beef production.

**Supplementary Information:**

The online version contains supplementary material available at 10.1007/s00484-026-03217-1.

## Introduction

Global demand for animal-based foods is projected to grow with current forecasts suggesting a significant increase in meat consumption throughout the 21st century (Jia et al. 2023). In particular, compared to 2022–2024, by 2033 beef production is expected to grow by about 11%, a rate that correlates closely with forecasted population dynamics (OECD/FAO 2025).

In this scenario, projected climate change is expected to exert major impacts on animal welfare and productivity (IPCC 2022; Thornton et al. 2022). Rising temperatures, changes in rainfall, and more frequent and intense extreme weather events represent the main climate features affecting directly or indirectly animal welfare and productivity (Lacetera 2019). Briefly, animal welfare is an attribute that can range from poor to excellent, it is compromised when an individual experiences difficulty in adapting to its environment, and may be assessed primarily through physiological and behavioural indicators (Broom 1991). However, an overlap exists between welfare and production-related measures, such as susceptibility to disease, mortality risk, growth performance, milk yield, and reproductive efficiency (Silanikove 2000). One of the primary direct ways global warming undermines animal welfare is through increased incidence and severity of heat stress (Thornton et al. 2022), which occurs when animals fail to dissipate sufficient internal or environmental heat to maintain thermal homeostasis; this, in turn, results in physiological and behavioural adaptations and adverse effects on health and production traits (Vitali et al. 2015; dos Santos et al. 2021).

To assess heat stress risk in beef cattle, Gaughan and colleagues (2008) developed the Heat-Load Index (HLI), which integrates air temperature, relative humidity, solar radiation, and wind speed into a single measure. Based on HLI, the same authors established thermal welfare categories according to empirical HLI thresholds (Gaughan et al. 2010). These thresholds were derived from observational studies across multiple commercial feedlots in Australia, encompassing both hot-dry Mediterranean and hot-humid subtropical climatic conditions. The dataset included over 12,000 steers representing 17 different genotypes, monitored across three consecutive summer seasons. Heat stress was quantitatively evaluated using panting scores, a validated physiological indicator of heat stress response (Mader et al. 2006), which assigns values on a scale from 0 (no stress) to 4.5 (extreme stress) based on distinct respiratory behaviours. The resulting classification comprises five heat stress categories: Comfort (HLI ≤ 70), Mild (70 < HLI ≤ 77), Moderate (77 < HLI ≤ 86), Severe (86 < HLI ≤ 96), and Extreme (HLI > 96).

Neither HLI nor the thermal welfare categories derived from HLI have been employed to estimate heat stress risks in beef cattle under different projected global socioeconomic conditions. The Shared Socioeconomic Pathways (SSPs) framework describes alternative global futures and was introduced by the Intergovernmental Panel on Climate Change (IPCC 2021). It combines social, economic, technological, and policy assumptions with greenhouse gas (GHG) emission projections (Riahi et al. 2017). Each SSP can be paired with Representative Concentration Pathways (RCPs), which outline standardized GHG concentration trajectories, together forming integrated SSP-RCP scenarios (O’Neill et al. 2017).

Within the SSPs framework, this study provides new information on projected increases of heat stress in beef cattle during the present century, using the HLI as a proxy for heat stress. The analysis, carried out both at the global and country level, established the seasonal distribution of heat stress categories for beef cattle over the climatological reference interval 1985–2014, and beyond this baseline, it incorporated future climate projections for 2026–2100.

## Materials and methods

### Source of climate data

The climate data utilized in this study were obtained from the NASA Earth Exchange (NEX) Global Daily Downscaled Projections (GDDP), hereafter referred to as the NEX-GDDP-CMIP6 archive (NASA Earth Exchange 2025). This dataset comprises outputs from General Circulation Models (GCMs) that have been downscaled through the Bias-Correction Spatial Disaggregation technique (Thrasher et al. 2022). The application of the Bias-Correction Spatial Disaggregation yields a spatially consistent, bias-adjusted dataset with a horizontal resolution of 0.25° × 0.25°, encompassing latitudinal ranges from 60°S to 90°N and thereby excluding Antarctica.

The present analysis is based on four fundamental daily climatic variables over the period 1985–2100: Near-surface air temperature (K), surface downwelling shortwave radiation (W/m²), mean near-surface wind speed (m/s), and near-surface relative humidity (%).

## Multi-model data selection and preprocessing

From the 35 GCMs included in the NEX-GDDP-CMIP6 archive, a subset of 27 models was selected based on the completeness of the required climate variables, thereby enabling a robust Multi-Model Ensemble (MME) analysis. MMEs are extensively recognized for their capacity to reduce biases and systematic errors inherent in individual model simulations, thus improving the robustness and credibility of climate projections (IPCC 2021). By aggregating outputs from multiple models, MMEs yield a more consistent and comprehensive representation of climate variability compared with single-model approaches (Dale et al. 2017; Slater et al. 2019; North et al. 2023). Detailed information regarding the selected GCMs is provided in the Online Resource Table S1.

Although the NEX-GDDP-CMIP6 archive has undergone rigorous quality assurance procedures (Thrasher et al. 2022), additional data checks were conducted to manage anomalies and ensure integrity. Specifically, placeholder values for missing data (1e + 20) were eliminated, negative values for wind speed and solar radiation, physically implausible, were set to zero, relative humidity values were constrained to the physically valid range (0-100%), and air temperatures below absolute zero (0 K) were excluded. Furthermore, a global terrestrial mask was applied to remove oceanic regions, which were not pertinent to this analysis.

All preprocessing steps and subsequent analyses were implemented using Python (v3.12), and runnable code listings are provided in a repository access in the Data and model availability statement.

## Seasonal and temporal aggregation

In alignment with the definition provided by the World Meteorological Organization (WMO), which designates a Climatological Normal (CliNo) as the mean of climatological parameters calculated over an uninterrupted 30-year period, this study established the global seasonal distribution of heat stress categories for beef cattle, derived from the HLI, over the climatological reference interval 1985–2014 (CliNo). This timeframe was selected as the baseline since, within the NEX-GDDP-CMIP6 archive, years following 2014 correspond to projected climate conditions rather than historical observations (Eyring et al. 2016), rendering them inappropriate for baseline purposes.

To construct the seasonal CliNo baseline, daily climate variables from each of the 27 selected GCMs were first averaged within individual models according to conventional seasonal groupings (Tuller 1990): December-February (DJF), March-May (MAM), June-August (JJA), and September-November (SON). The MME seasonal mean was then obtained by applying equal weighting and averaging (Noce et al. 2020; Wu et al. 2020) across the 27 models.

Beyond the seasonal CliNo baseline, the analysis incorporated future climate projections for 2026–2100 under the four SSPs: SSP1-2.6, SSP2-4.5, SSP3-7.0, and SSP5-8.5. These scenarios encompass a broad range of potential socioeconomic trajectories and associated GHG emissions. SSP1-2.6 (SSP1 with RCP2.6) reflects a sustainable development pathway with low emissions, stabilizing radiative forcing near 2.6 W/m^2^ by 2100. SSP2-4.5 (SSP2 with RCP4.5) represents an intermediate pathway with moderate emissions, leading to stabilization around 4.5 W/m^2^ by the end of the century. SSP3-7.0 depicts a medium-to-high emissions scenario driven by regional competition, yielding radiative forcing of approximately 7.0 W/m^2^ by 2100. Finally, SSP5-8.5 characterizes a fossil-fuel-dependent pathway associated with very high emissions, resulting in radiative forcing of 8.5 W/m^2^ by 2100 (Eyring et al. 2016; Riahi et al. 2017).

Following the same methodology applied to the seasonal CliNo baseline, seasonal climatological means for each SSP scenario were computed over three successive 25-year intervals: 2026–2050, 2051–2075, and 2076–2100.

## Seasonal HLI computation

The HLI was derived from the MME seasonal mean climate variables using a two-step procedure. In the first step, black globe temperature, absent from GCM outputs, was estimated from near-surface air temperature and incident shortwave solar radiation by applying an empirical formulation that accounts for both thermal and radiative environmental influences (Carvajal et al. 2021). In the second step, the estimated black globe temperature, together with relative humidity and wind speed, was incorporated into the HLI equation as formulated by Gaughan et al. (2008 and 2010).

The HLI was calculated on a global scale with a spatial resolution of 0.25° × 0.25°, for both the CliNo baseline period and the projected seasonal climates under the four SSP scenarios. The full set of equations employed in the computation is provided in the Online Resource Equation S1.

## Seasonal mapping of beef cattle thermal welfare categories

Seasonal maps of thermal welfare categories were produced by classifying, at each 0.25° land grid cell, the seasonal mean HLI fields (DJF, MAM, JJA, SON) derived from the equal-weighted MME. Following the thresholds defined above (Gaughan et al. 2008; 2010), HLI values were assigned to five heat stress categories: Comfort (HLI ≤ 70), Mild (70 < HLI ≤ 77), Moderate (77 < HLI ≤ 86), Severe (86 < HLI ≤ 96) and Extreme (HLI > 96). The same classification was applied to the CliNo baseline and projected seasonal climates to ensure comparability across periods and scenarios. The generated maps depict the seasonal distribution of HLI-based heat stress categories for beef cattle and highlight regions where heat stress risk is projected to rise or decline relative to the 1985–2014 baseline under each SSP.

All cartography was generated in Python (v3.12) and runnable code listings are provided in a repository access in the Data and model availability statement.

### Global and country-level analysis of expansion of extreme heat stress areas

In addition to the seasonal global maps of heat stress categories, changes in the land area exposed to extreme heat stress (HLI > 96) at both global and country level were quantified. Specifically, national boundaries were taken from the publicly available layer of the Natural Earth dataset (www.naturalearthdata.com) and used to derive country-level summaries on the 0.25° × 0.25° CliNo and projection grids. Specifically, for each grid cell, the corresponding land area (km^2^) was computed following the formulation described by Yang et al. (2024). All land grid cells with seasonal mean HLI > 96 were flagged as belonging to the extreme heat stress category. For each country, and for the globe as a whole, we then summed the areas of all flagged cells and divided this by the total terrestrial area, obtaining the fraction of land area exposed to extreme HLI. Changes in exposure were expressed as percentage-point (pp) differences between each of the three projected 25-year intervals period and the CliNo baseline. These metrics were summarised in a table as seasonal changes in global land area expose to extreme heat stress relative to the CliNo period, and visualised in the supplementary materials as maps of pp changes by country for the three projected 25-year intervals.

All cartography and area-based tabulations were generated in Python (v3.12). Runnable code is available in the repository referenced in the Data and model availability statement.

## Results

### Beef cattle thermal welfare categories relative to CliNo

**Fig. 1:**
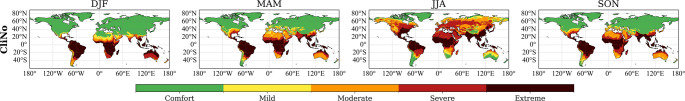
Global seasonal distribution of the Heat-Load Index (HLI) expressed in terms of heat stress categories established for beef cattle and averaged over the climatological reference period (1985–2014, CliNo). Data derived from the multi-model ensemble mean of 27 models from the NEX-GDDP-CMIP6 dataset at a 0.25° x 0.25° spatial resolution. Each panel refers to one of the four meteorological seasons: DJF (December-February), MAM (March-May), JJA (June-August), and SON (September-November). The colour represents the five heat stress categories: green (Comfort) yellow (Mild), orange (Moderate), red (Severe) and dark red (Extreme)

Figure 1 illustrates global seasonal distribution of the heat stress categories established for beef cattle based on HLI over the climatological reference period 1985-2014 (CliNo).

During austral summer (DJF), extensive regions within tropical and subtropical latitudes showed HLI ranging from severe (86–96) and extreme (> 96) heat stress. Conversely, large portions of the Northern Hemisphere remain within comfort zones due to cooler winter conditions. Transitioning into boreal spring (MAM), the risk of heat stress ranging from moderate to extreme, expands northward into southern Europe, southern Asia, and the southern United States.

Boreal summer (JJA) presents the most pronounced risk of heat stress into the Northern Hemisphere, with large areas of North America, Europe, Asia, and North Africa transitioning into moderate-to-extreme heat stress categories. Equatorial and subtropical regions are characterized by conditions associated with heat stress ranging from severe to extreme. Austral spring (SON) shows a partial alleviation of the heat stress risk across northern latitudes, although tropical and subtropical regions continue to face serious heat-related welfare challenges.

### Projected beef cattle thermal welfare conditions for the early-century (2026–2050)

**Fig. 2:**
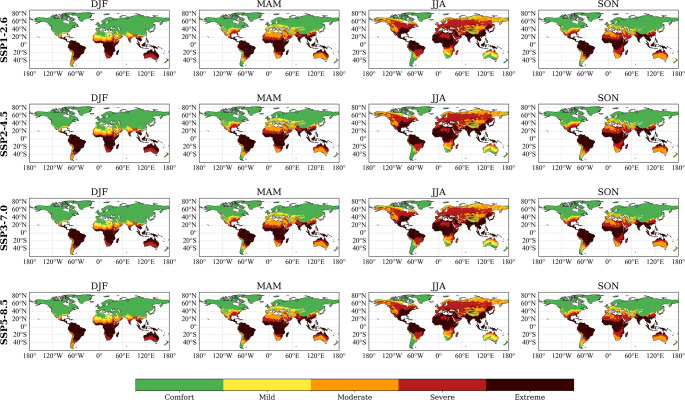
Seasonal distribution of the Heat-Load Index (HLI) expressed in terms of heat stress categories established for beef cattle under different climate scenarios for the period 2026–2050. Each row corresponds to a different Shared Socioeconomic Pathway (SSP1-2.6, SSP2-4.5, SSP3-7.0, and SSP5-8.5), while each column shows a meteorological season (DJF: December-February; MAM: March-May; JJA: June-August; SON: September-November). The maps are derived from the multi-model ensemble mean of 27 models from the NEX-GDDP-CMIP6 dataset at a 0.25° x 0.25° spatial resolution. The colour scale represents the five heat stress categories: green (Comfort), yellow (Mild), orange (Moderate), red (Severe) and dark red (Extreme)

Figure 2 illustrates global seasonal distributions of the heat stress categories based on HLI and projected for the period 2026-2050, under the four SSPs.

Under SSP1-2.6, risk of severe (86–96) and extreme (> 96) heat stress prominently affect equatorial and subtropical regions, particularly visible across central Africa, southern Asia, northern Australia, and parts of South America during austral (DJF) and boreal summer (JJA). In contrast, higher-latitude regions in the Northern Hemisphere clearly remain between comfort (≤ 70) and risk of mild stress (70–77) throughout the year.

Under SSP2-4.5, risk of moderate (77–86) to severe (86–96) heat stress expands during boreal summer (JJA), particularly in southeast Europe, southern North America, central Asia, and Australia. Equatorial areas are projected to experience consistent conditions associated to severe or extreme heat stress across all seasons, highlighting their persistent vulnerability.

Under SSP3-7.0, risk of heat stress ranging from severe and extreme remains pronounced primarily in equatorial and subtropical regions, with particularly marked intensity detectable during austral (DJF) and boreal summer (JJA). Risk of moderate and severe heat stress conditions slightly expand northward in the boreal summer, notably affecting southern parts of North America, Europe, and Asia, while higher northern latitudes would continue to experience predominantly comfortable or mild heat stress conditions. Persistent severe conditions of heat stress are projected across tropical and subtropical regions, highlighting limited seasonal relief.

Finally, under the SSP5-8.5 scenario, characterized by the highest emission trajectory, the probability of severe or extreme heat stress slightly expands, especially during boreal summer (JJA), affecting southeast Europe, central and southern Asia, southern North America, and subtropical regions of South America and Africa. Even during transitional seasons (MAM and SON), moderate to severe heat stress persists in many subtropical and tropical regions, suggesting lack of potential relief periods during the year.

### Projected beef cattle thermal welfare conditions for the mid-century (2051–2075)

**Fig. 3:**
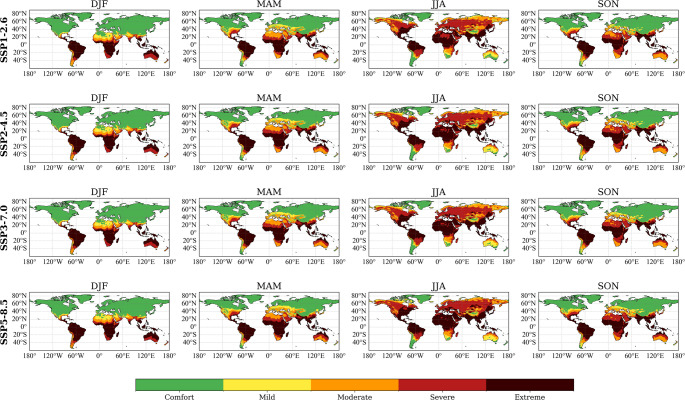
Seasonal distribution of the Heat-Load Index (HLI) expressed in terms of heat stress categories established for beef cattle under different climate scenarios for the period 2051–2075. Each row corresponds to a different Shared Socioeconomic Pathway (SSP1-2.6, SSP2-4.5, SSP3-7.0, and SSP5-8.5), while each column shows a meteorological season (DJF: December-February; MAM: March-May; JJA: June-August; SON: September-November). The maps are derived from the multi-model ensemble mean of 27 models from the NEX-GDDP-CMIP6 dataset at a 0.25° x 0.25° spatial resolution. The colour scale represents the five heat stress categories: green (Comfort), yellow (Mild), orange (Moderate), red (Severe) and dark red (Extreme)

Figure 3 shows global seasonal distributions of the heat stress categories based on HLI and projected for the period 2051-2075, under the four SSPs.

Under the lowest-emission scenario (SSP1-2.6), severe (86–96) to extreme (> 96) categories remain clearly concentrated in tropical and subtropical regions, primarily during austral (DJF) and boreal summer (JJA). Regions such as central Africa, India, Southeast Asia, northern Australia, and significant areas of South America are consistently projected to experience moderate (77–86) or extreme heat stress conditions. In contrast, temperate and high-latitude zones will predominantly experience comfort (≤ 70) to mild heat stress (70–77), especially during boreal winter (DJF) and autumn (SON).

Compared to SSP1-2.6, the intermediate-emission scenario (SSP2-4.5) indicates an increase of the spatial extent of conditions causing moderate or extreme heat stress. During boreal summer, the expansion of conditions of severe or extreme heat stress is particularly evident in the Mediterranean basin, southern regions of North America, central Asia, and notably larger portions of Australia. Transitional seasons (MAM and SON) also show a moderate increase in heat stress severity, primarily affecting subtropical and mid-latitude areas.

Under scenario SSP3-7.0, heat stress conditions slightly expand during JJA, notably in parts of central Asia. Equatorial regions continue experiencing persistent severe to extreme heat stress throughout the year. However, compared to the SSP2-4.5 scenario, the increase in geographical extent of severe and extreme heat stress categories are modest and not particularly pronounced.

Under the highest-emission scenario (SSP5-8.5), there is a substantial expansion of severe and extreme heat stress categories at global level, most pronounced during boreal summer (JJA). Large areas, particularly southern North America, southern Europe, central and southern Asia, and extensive subtropical regions of Africa, South America, and Australia, are exposed to prolonged periods of risk of heat stress ranging from severe and extreme. Finally, transitional seasons (MAM and SON) reveal extensive persistence of moderate to severe heat stress conditions across large subtropical zones.

### Projected beef cattle thermal welfare conditions for the late-century (2076–2100)

**Fig. 4:**
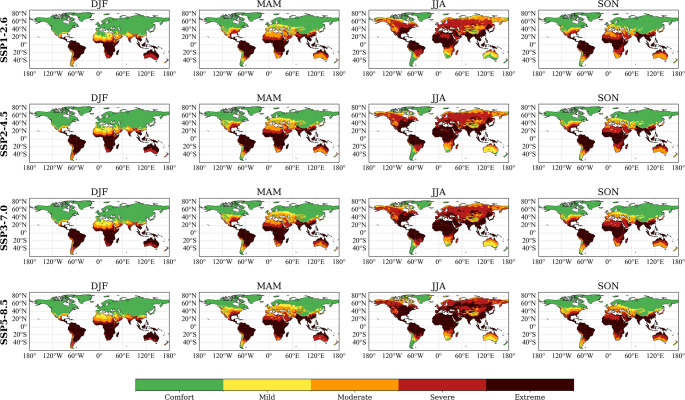
Seasonal distribution of the Heat-Load Index (HLI) expressed in terms of heat stress categories established for beef cattle under different climate scenarios for the period 2076–2100. Each row corresponds to a different Shared Socioeconomic Pathway (SSP1-2.6, SSP2-4.5, SSP3-7.0, and SSP5-8.5), while each column shows a meteorological season (DJF: December-February; MAM: March-May; JJA: June-August; SON: September-November). The maps are derived from the multi-model ensemble mean of 27 models from the NEX-GDDP-CMIP6 dataset at a 0.25° x 0.25° spatial resolution. The colour scale represents the five heat stress categories: green (Comfort), yellow (Mild), orange (Moderate), red (Severe) and dark red (Extreme)

Figure 4 shows global seasonal distributions of the heat stress categories based on HLI and projected for the period 2076-2100, under the four SSPs.

Under SSP1-2.6, categories of severe (86–96) and extreme (> 96) heat stress remain concentrated in equatorial, tropical, and subtropical regions. During boreal (JJA) and austral summer (DJF), extensive areas within central Africa, northern Australia, India, Southeast Asia, and South America remain persistently subjected to risk of heat stress between moderate (77–86) and extreme heat stress. Temperate regions generally remain in comfort (≤ 70) or mild stress (70–77) categories during winter (DJF) and transitional seasons (MAM and SON).

Compared to SSP1-2.6, the SSP2-4.5 scenario projects a considerable expansion of severe or extreme heat stress areas, particularly in the boreal summer (JJA). Extensive regions in North America, southern Europe, East Asia, and South America are projected to experience widespread moderate to extreme heat stress conditions, with substantial areas of severe to extreme stress also evident during spring (MAM) and autumn (SON).

The SSP3-7.0 scenario further increases the gravity of heat stress globally, markedly extending severe and extreme heat stress categories into higher latitudes. During JJA, nearly all temperate regions, including extensive parts of North America, Europe, central Asia, and southern parts of South America and Australia, are projected to experience predominantly severe or extreme heat stress. Even during transitional seasons (MAM and SON), extensive regions remain within moderate to severe heat stress categories, significantly constraining periods of thermal comfort.

The most extreme emissions scenario, SSP5-8.5, demonstrates marked global intensification and expansion of severe and extreme heat stress categories. Nearly all continents are projected as severely affected during boreal (JJA) and austral summer (DJF), with extensive areas experiencing sustained severe to extreme heat stress conditions throughout most of the year. Regions typically temperate, including large portions of North America, Europe, central Asia, and southern hemispheric mid-latitudes, are projected to undergo dramatic increases in severe and extreme heat stress even during spring and autumn.

### Global and country changes in extreme heat stress exposure

Table 1 summarises, for each SSP and for the three successive 25-year intervals, the increase in global land area where beef cattle are potentially exposed to extreme HLI conditions. The increase was expressed as absolute pp differences relative to the CliNo period.

Even under baseline conditions, roughly one quarter to one third of the global terrestrial surface experienced seasonal mean HLI values falling within the extreme heat stress category, with JJA showing the highest share. Across all SSPs, the area exposed to extreme conditions is projected to expand in every season and time slice, with boreal summer exhibiting the most pronounced changes. In JJA, indeed, the share of global land with HLI > 96 increases from about 31% in the CliNo baseline to nearly 40% by 2026–2050 and to almost 60% by 2076–2100 under SSP5-8.5, whereas lower-emission SSPs show smaller, but still non-negligible, expansions.

**Fig. 5:**
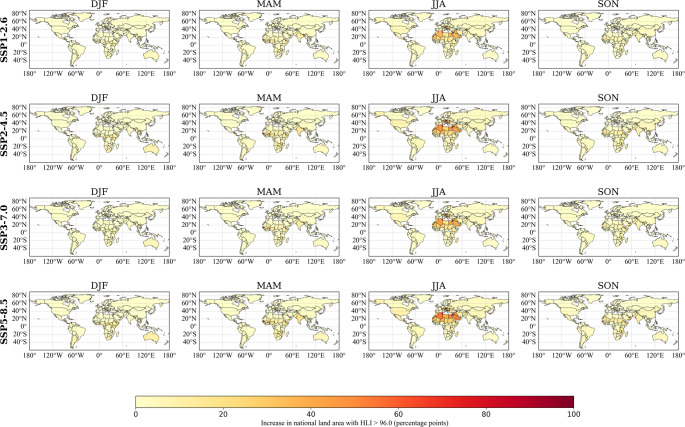
Seasonal increase in national land area exposed to extreme heat stress for 2026–2050 relative to the climatological normal (CliNO, 1985–2014). Columns show the four seasons (DJF, MAM, JJA, SON), and rows correspond to the four SSP scenarios (SSP1-2.6, SSP2-4.5, SSP3-7.0, SSP5-8.5). Colours indicate the change in the fraction of national land area with HLI > 96, expressed as percentage-point differences from the CliNO baseline

**Fig. 6:**
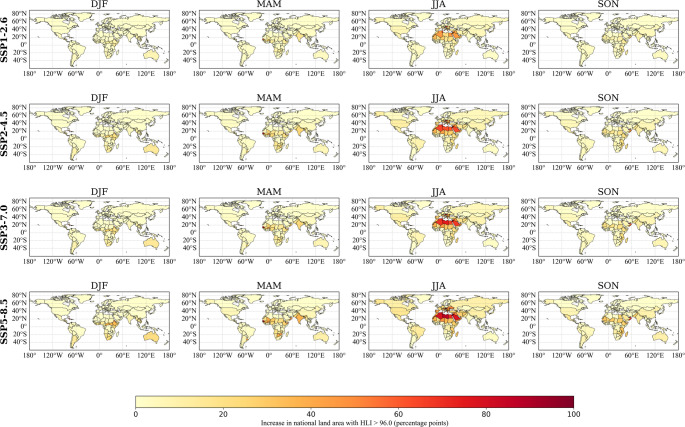
Seasonal increase in national land area exposed to extreme heat stress for 2051–2075 relative to the climatological normal (CliNO, 1985–2014). Columns show the four seasons (DJF, MAM, JJA, SON), and rows correspond to the four SSP scenarios (SSP1-2.6, SSP2-4.5, SSP3-7.0, SSP5-8.5). Colours indicate the change in the fraction of national land area with HLI > 96, expressed as percentage-point differences from the CliNO baseline

**Fig. 7:**
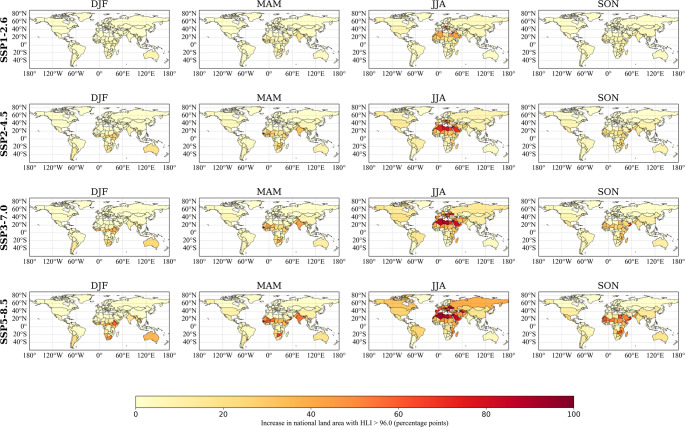
Seasonal increase in national land area exposed to extreme heat stress for 2076–2100 relative to the climatological normal (CliNO, 1985–2014). Columns show the four seasons (DJF, MAM, JJA, SON), and rows correspond to the four SSP scenarios (SSP1-2.6, SSP2-4.5, SSP3-7.0, SSP5-8.5). Colours indicate the change in the fraction of national land area with HLI > 96, expressed as percentage-point differences from the CliNO baseline

Furthermore, to address spatial heterogeneity, country-level changes in the fraction of national land area exposed to extreme heat stress level (pp differences from CliNo) were established and presented in the appendix (Figs. 5, 6 and 7) for the three projected periods and the four SSP scenarios. Across scenarios, the maps highlight a strong seasonal signal, with the most pronounced national increases concentrated in JJA. By late century under higher-emission pathways, the largest expansions appear clustered in hot and arid-to-semi-arid belts, particularly across North Africa and the Middle East, with additional hotspots in parts of Sub-Saharan Africa and South Asia. In contrast, many higher-latitude countries show comparatively modest changes, especially under SSP1-2.6 and in the transitional seasons. Taken together, the country maps indicate that the projected expansion of the extreme heat stress category will affect low-income regions with already warm climates, which include large portions of Sub-Saharan Africa and parts of South and West Asia.

**Table 1:** Seasonal changes in global land area exposed to extreme heat stress (HLI > 96) under different SSP scenarios, expressed as absolute percentage-point (pp) differences relative to the CliNo period

Season	Time range	CliNo extreme heat stress share (%)	SSP1-2.6 (pp)	SSP2-4.5 (pp)	SSP3-7.0 (pp)	SSP5-8.5 (pp)
DJF	2026–2050	25.4	1.1	1.6	1.2	2.0
	2051–2075		1.5	2.7	2.8	4.2
	2076–2100		1.4	3.5	4.8	7.0
MAM	2026–2050	27.2	1.7	2.5	1.8	3.0
	2051–2075		2.3	4.1	4.1	6.2
	2076–2100		2.3	5.4	6.9	9.8
JJA	2026–2050	31.2	5.1	6.9	5.8	8.3
	2051–2075		6.7	10.6	11.1	15.3
	2076–2100		6.6	13.2	17.6	26.6
SON	2026–2050	27.2	1.8	2.5	2.0	3.1
	2051–2075		2.4	4.2	4.4	6.5
	2076–2100		2.3	5.4	7.5	12.0

## Discussion

Even under scenarios characterized by low to moderate GHG emissions, the study projected a global expansion of the HLI categories associated with risk of severe or extreme heat stress in beef cattle. While numerous non-climatic factors, such as housing, genetic traits, nutrition, production level, health status, and age, also influence the severity of heat stress (Brown-Brandl 2018), our results corroborate the broader consensus that climate change is likely to exert substantial negative impacts on livestock welfare. Moreover, the findings are consistent with global-scale analyses, which indicate escalating thermal risks in tropical and subtropical regions where livestock are already frequently exposed to elevated temperatures (Carvajal et al. 2021; Thornton et al. 2022; North et al. 2023).

From a quantitative perspective, the comparison between the baseline and late-century periods under high-emission pathways suggests that the fraction of global land projected to experience extreme summer HLI approximately doubles. Importantly, even low-emission scenarios are associated with a substantial expansion of areas exposed to extreme heat stress, implying that climate mitigation alone may be insufficient to prevent widespread increases in thermal stress risk for livestock systems.

Despite the different approach, our findings are broadly consistent with the Temperature-Humidity Index (THI)-based projections of Thornton et al. (2021). However, notable differences in the spatial distribution and intensity of projected hotspots emerge, because HLI extends THI by explicitly incorporating solar radiation and wind speed. These two variables are two key drivers of heat stress in grazing and open-housing systems, and, if considered in association with temperature and humidity, allow a more accurate characterization of livestock exposure, particularly in hot, dry, and high-radiation environments.

Our findings are also in line with those from previous studies indicating that the effects of climate change on cattle meat yield will be more severe in low-income countries (Liu et al. 2024). In contrast, regions located at higher latitudes are predicted to experience a decrease in cold-related stress, which would accompany an increase of arable land (Hu et al. 2024). The projected adverse effects of climate change on the production of meat and other animal-derived foods therefore generate a tension that might make it more challenging to satisfy the projected increase in global demand. Because population and demand growth will be higher in the South, production is likely to remain or even expand in the Global North, as environmental constraints imposed by climate change severely limit the feasibility of scaling up production, especially at lower latitudes. If, on the one hand, the projected increase in high-latitude areas may help offset at least partially the reduction in agricultural productivity observed elsewhere (Godde et al. 2021), on the other hand the reallocation from the Global South to the Global North would imply worse terms for trade for lower-income countries. This, in turn, would widen economic disparities between areas, a pattern that is argued to make a sustainable economic development path more difficult to achieve (Islam 2015).

Beef cattle are commonly managed under pastoral, feedlot, or semi-intensive systems, which expose them to variable and stressful climatic conditions (Gaughan and Cawdell-Smith 2015; Rojas-Downing et al. 2017; Jack et al. 2022). Compared to dairy, beef cattle exhibit greater tolerance to high HLI values, primarily because of their lower endogenous metabolic heat production and more pronounced genetic diversity among breeds (Nardone et al. 2010; Rhoads et al. 2013). However, despite their relative tolerance, beef cattle remain vulnerable to heat stress, which notably induces an immediate reduction in feed intake (Thornton et al. 2022). Shortly after feed intake decline, cattle initiate thermoregulatory responses, including panting, sweating, and augmented water intake (Gaughan et al. 2010; Wankar et al. 2024). However, heat stress extends beyond the direct suppression of appetite, and indirectly affects key metabolic pathways, digestive function, and efficiency of nutrient absorption (Sejian et al. 2018; Wankar et al. 2019). These behavioural and physiological adjustments redirect energy from productive processes toward maintaining homeostasis, thereby impairing growth performance, reducing daily weight gain, and increasing the risk of diseases and mortality (Nardone et al. 2010; Marchesini et al. 2018; Liu et al. 2024).

The projected magnitude of the HLI increases leads one to support findings and considerations from other authors who indicated that, while necessaries, adaptation strategies are unlikely to fully reverse the effects of heat stress conditions (Palandri et al. 2025). However, regardless of the scenario that unfolds, substantial and widespread adaptation efforts will be required in the coming decades to prevent profound deterioration of animal welfare and severe production losses in beef cattle, as well as in the other food-producing animals (Segnalini et al. 2013; Thornton et al. 2022). With regard to beef cattle, effective strategies will need to include climate-smart management of pasture (Montazar and Afshar 2025), provision of natural or artificial shading, selective breeding for heat tolerance, and optimized feeding schedules (Gaughan et al. 2019). Moreover, implementing technologies like precision agriculture and livestock farming can optimize resource use, even if this requires significant investment and proper training for farmers (Nsabiyeze et al. 2025).

To effectively support adaptation strategies, policymakers should thus prioritize the formulation of region-specific guidelines and introduce financial incentives that encourage the adoption of climate-smart livestock practices in areas most vulnerable to heat stress (van Asseldonk et al. 2023). In parallel, governments ought to invest in research and development initiatives aimed at raising heat-tolerant livestock breeds, thereby fostering the long-term sustainability of production systems (Cheruiyot et al. 2022). In addition to public intervention, or in collaboration with it, market solutions may contribute to mitigate the effects of global warming. Insurance companies, for example, are introducing forms of index-based insurance against heat stress in animals whereby farmers are compensated if certain indicators, such as temperature, pass pre-defined thresholds, independently of the actual effects of those high temperatures (Bielza Diaz-Caneja et al. 2008). This coverage may alleviate economic losses and provide funds for further investment in sustainability and resilience as well as limit distortion such as moral hazard and adverse selection. Climate-related risks, however, are not idiosyncratic, i.e. specific to a given farm: they are instead highly correlated, for example within geographical areas. Negative events (e.g. a rise in temperature) would require an insurance company to compensate all affected clients at once, and this may be financially sustainable only by charging high premia, thus likely limiting uptake. Uptakes of these instruments, especially in the global south, have indeed been low (Binswanger-Mkhize 2012). Reinsurance schemes from specialized companies or through government interventions may address this market friction, at least to some extent.

In sum, the livestock sector faces an unprecedented dilemma. On the one hand, it is under pressure to guarantee at the same time high standards of animal welfare and output expansion to meet nutritional and economic expectations. On the other hand, it must operate with large areas of the planet that will experience increasingly restrictive ecological boundaries. Addressing this contradiction requires transformative strategies, including investments in climate-resilient agricultural practices, the reduction of food loss and waste, and the promotion of dietary diversification. Without such interventions, the negative effects of climate change are likely to prevent animal agriculture from meeting future global demand, with profound implications for food security and environmental sustainability.

## Supplementary Information

Below is the link to the electronic supplementary material.

Supplementary Material 1

## Data Availability

Climate inputs are publicly available from the NASA Earth Exchange NEX-GDDP-CMIP6 archive (NASA Earth Exchange 2025; accessed on 23 September 2025). The derived datasets used to produce the figures in this study (seasonal Heat-Load Index at 0.25° resolution, global, baseline 1985-2014 and CMIP6 projections 2026-2100; multi-model ensemble) are openly available at Figshare: 10.6084/m9.figshare.30136138.v2. The analysis scripts are openly available in the GitHub repository HLI-BeefCattle-Welfare-Seasonal-Projections:https://github.com/Giampiero-G/HLI-beefcattle-welfare-seasonal-projections.git (accessed on 18 December 2025).
