# Supplementary material for: Integrating climate scenarios and thermal welfare thresholds to project future heat stress risk in beef cattle
Source: Int J Biometeorol. 2026 May 7;70(5):155. doi: 10.1007/s00484-026-03217-1 (PMC13152964; doi:10.1007/s00484-026-03217-1)
Supplement: Supplementary file 1 — Supplementary Material 1 [file 484_2026_3217_MOESM1_ESM.docx]

***International Journal of Biometeorology***

**Integrating climate scenarios and thermal welfare thresholds to project future heat stress risk in beef cattle**

Giampiero Grossi *a*, Andrea Vitali *a*, Nicola Lacetera *b*, Chiara Rossi *a*, Nicola Lacetera *a*

*^a^*Department of Agriculture and Forest Sciences, University of Tuscia, Via San Camillo De Lellis, 01100, Viterbo, Italy

*^b^*Department of Economic Sciences, University of Bologna, Piazza Scaravilli 2, Bologna, Italy

Corresponding author: Giampiero Grossi. [g.grossi@unitus.it](mailto:g.grossi@unitus.it)

**Table S1** List of the NEX-GDDP-CMIP6 global climate models involved in the study.

| **No.** | **General Circulation Models** | **Modelling centre** |
| --- | --- | --- |
| 1 | ACCESS-CM2 | Commonwealth Scientific and Industrial Research Organisation (CSIRO), Australia |
| 2 | ACCESS-ESM1-5 | Commonwealth Scientific and Industrial Research Organisation (CSIRO), Australia |
| 3 | CanESM5 | Canadian Centre for Climate Modelling and Analysis (CCCma), Canada |
| 4 | CESM2 | National Centre for Atmospheric Research (NCAR), USA |
| 5 | CMCC-CM2-SR5 | Euro-Mediterranean Centre on Climate Change (CMCC), Italy |
| 6 | CMCC-ESM2 | Euro-Mediterranean Centre on Climate Change (CMCC), Italy |
| 7 | CNRM-CM6-1 | Centre National de Recherches Météorologiques (CNRM), France |
| 8 | CNRM-ESM2-1 | Centre National de Recherches Météorologiques (CNRM), France |
| 9 | EC-Earth3 | EC-Earth Consortium |
| 10 | EC-Earth3-Veg-LR | EC-Earth Consortium |
| 11 | FGOALS-g3 | Institute of Atmospheric Physics, Chinese Academy of Sciences, China |
| 12 | GFDL-ESM4 | Geophysical Fluid Dynamics Laboratory (GFDL), USA |
| 13 | GISS-E2-1-G | Goddard Institute for Space Studies (GISS), NASA, USA |
| 14 | IITM-ESM | Indian Institute of Tropical Meteorology (IITM), India |
| 15 | INM-CM4-8 | Institute of Numerical Mathematics (INM), Russia |
| 16 | INM-CM5-0 | Institute of Numerical Mathematics (INM), Russia |
| 17 | IPSL-CM6A-LR | Institute Pierre-Simon Laplace (IPSL), France |
| 18 | KACE-1-0-G | National Institute of Meteorological Sciences (NIMS), South Korea |
| 19 | MIROC6 | Japan Agency for Marine-Earth Science and Technology (JAMSTEC), Japan |
| 20 | MIROC-ES2L | Japan Agency for Marine-Earth Science and Technology (JAMSTEC), Japan |
| 21 | MPI-ESM1-2-HR | Max Planck Institute for Meteorology (MPI-M), Germany |
| 22 | MPI-ESM1-2-LR | Max Planck Institute for Meteorology (MPI-M), Germany |
| 23 | MRI-ESM2-0 | Meteorological Research Institute (MRI), Japan |
| 24 | NorESM2-LM | Norwegian Climate Centre (NCC), Norway |
| 25 | NorESM2-MM | Norwegian Climate Centre (NCC), Norway |
| 26 | TaiESM1 | Research Centre for Environmental Changes, Academia Sinica, Taiwan |
| 27 | UKESM1-0-LL | Met Office Hadley Centre, United Kingdom |

***HLI equation (S1)***

The HLI CliNO map, was developed using the equation provided by Gaughan et al., (2008, 2010), which is as follows:

For *T_bg_*>25°C:

${HLI}_{T_{bg}>25}= 8.62+\left( 0.38 \times hurs \right)+ \left( 1.55 \times T_{bg} \right)-\left( 0.5 \times sfcWind \right)+[e^{2.4-sfcWind}]$ [1]

For T_bg_<25°C:

${HLI}_{T_{bg}<25}= 10.66+\left( 0.28 \times hurs \right)+ \left( 1.3 \times T_{bg} \right)-sfcWind$ [2]

Where T_bg_ is calculated as:

$T_{bg}=1.33\times tas\_c-2.65\times{tas\_c}^{0.5}+3.21\log\left( rsds+1 \right)+3.5$ [3]

The sigmoid function for Black Globe Temperature (T_bg_) was determined by:

$S\left( T_{bg} \right)=\frac{1}{(1+exp\left( -\frac{\left( T\_bg-25 \right)}{2.25} \right)}$ [4]

and the final HLI was computed as:

$HLI=S\left( T_{bg} \right)\times{HLI}_{T_{bg}>25}+\left( 1-S\left( T_{bg} \right) \right)\times{HLI}_{T_{bg}<25}$ [5]

Where:

*hurs* = near-surface relative humidity (%)

*T_bg_* = black globe temperature (°C). Equation [4], as provided by Carvajal et al., (2021), smooths the transition from air temperature to Black Globe Temperature.

*sfcWind* = daily mean near-surface wind speed (m s⁻¹),

*tas_c* = near-surface air temperature in °C

*rsds* = surface downwelling shortwave radiation (W m⁻²)
